# Synthetic estrogen and progestin effects on the myogenic program following damage in C2C12 murine myoblasts

**DOI:** 10.14814/phy2.70886

**Published:** 2026-04-26

**Authors:** Mai Wageh, Michael Kamal, Gianni Parise

**Affiliations:** ^1^ Department of Kinesiology McMaster University Hamilton Ontario Canada

**Keywords:** electrical stimulation, hormones, muscle damage, myoblasts, oral contraceptives

## Abstract

Females exhibit distinct responses to exercise‐induced muscle damage, possibly due to circulating hormone profiles. Despite the widespread use of oral contraceptives (OC) among females, the muscle damage and repair response during synthetic hormone use remains unknown. This study investigated how ethinyl estradiol (EE) and synthetic progestins [representing the different OC generations: medroxyprogesterone acetate (MPA), OC1; levonorgestrel (LNG), OC2; desogestrel (DSG), OC3; and drospirenone (DRP), OC4] influence C2C12 myoblast behavior. We examined proliferation, differentiation, and migration at baseline and following electrical pulse stimulation (EPS). While MPA and LNG alone increased myoblast proliferation (~19%; *p* < 0.05), their combination with EE (creating OC1 and OC2, respectively) reduced proliferation (15%–17%; *p* < 0.05) versus the vehicle control (VEH). All OC formulations significantly impaired migration (46%–61%; *p* < 0.001) without affecting differentiation. Post‐EPS, only OC1 and OC2 conditions showed elevated creatine kinase alongside upregulated early myogenic proteins and modulation of hormone receptors. These results indicate that synthetic sex hormones in OCs can significantly alter myoblast responses to damaging stimuli in a generation‐specific manner. OC use may influence muscle repair processes differently than natural hormonal profiles, highlighting the need for further investigation into how contraceptive formulations affect exercise recovery in women.

## Graphical abstract

The effect of oral contraceptives (OCs) on muscle repair is unknown. Using electrical pulse stimulation to simulate damage in C2C12 myoblasts treated with four OC generations (MPA, LNG, DSG, DRP combined with ethinyl estradiol), we assessed proliferation, differentiation, migration, creatine kinase, myogenic proteins, and hormone receptor protein abundance. All OC formulations impaired migration, first‐ and second‐generation OCs reduced proliferation and elevated damage markers post‐stimulation, and none affected differentiation, indicating that synthetic sex hormones may alter repair responses in a generation‐specific manner.

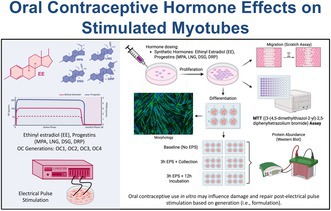

## 1 INTRODUCTION

Skeletal muscle is an essential tissue for movement and metabolic function and is regulated by a complex network of endogenous and exogenous factors. Part of this regulation involves the activity of muscle stem cells, or satellite cells (SC), that activate, proliferate, and differentiate to repair damaged muscle tissue (Snijders et al., 2015). Males tend to have a rapid and pronounced expansion of the SC pool following injury, while females have been shown to exhibit a less robust response (Fortino et al., 2022). It has been suggested that this discrepancy is due to estrogen offering a protective advantage to females, therefore limiting damage and the subsequent SC response (Collins et al., 2019; Kendall & Eston, 2002; Persky et al., 2000). The mechanistic basis behind this theory has not yet been revealed, but there is literature to suggest that estrogen aids in membrane stabilization as well as the blunting of the inflammatory response to exercise (Bombardier et al., 2009; D'Eon et al., 2008; Enns et al., 2008; Komulainen et al., 1999; Stupka et al., 2000).

As a highly plastic tissue, skeletal muscle may be influenced by exogenous hormones, such as oral contraceptive (OC) use or hormone replacement therapy (HRT). 75% of Canadian women will, at some point in their lifetime, use an OC, making it the most prescribed drug for premenopausal women (Rotermann et al., 2015). OCs are pharmaceutical drugs containing the synthetic forms of estrogen (known as ethinyl estradiol; EE) and progesterone (termed progestins) (Cooper & Patel, 2024). There are currently four generations of OCs, each containing a distinct synthetic progestin with slightly different mechanisms of action. Most commonly, first‐generation OCs (OC1) are often comprised of EE and norethindrone or medroxyprogesterone acetate; second‐generation OCs (OC2) include EE and levonorgestrel; third‐generation OCs (OC3) combine EE and norgestimate or desogestrel; and fourth‐generation OCs (OC4) include a mix of EE and dienogest or drospirenone (Allen, 2022). Similar to natural estrogen and progesterone, synthetic hormones exert their effects primarily through their receptors (estrogen receptor, ER; progesterone receptor, PR). Receptor function can be modulated by endogenous or synthetic hormone levels, the hormone's affinity for the receptor, and interactions with other hormones or compounds, which may either inhibit or enhance signaling (Evans, 2017). However, the direct effects of OC formulations on receptor function and the resulting hormonal responses remain poorly understood.

As a result of their varying chemical compositions, each OC generation may invoke different effects on skeletal muscle. Investigations examining the effect of OC ingestion over longer periods show that EE + gestogen (a third‐generation progestin) induces significantly greater increases in type I muscle fiber area with resistance training than naturally cycling individuals (Evans, 2017). Another study comparing “high‐androgen” (EE and levonorgestrel) and “anti‐androgenic” (estradiol + cyproterone acetate – first‐generation progestin) OC users showed significantly greater increases in fat‐free mass and muscle strength in the high‐androgenic OC group after training despite no differences in VO_2_max (Darney, 1995). These adaptations may be, in part, due to an increase in SC content in type II myofibers, as seen after 10 weeks of training in Danish OC users consuming EE + levonorgestrel (Sitruk‐Ware, 2004). Rodent models using EE alone demonstrate faster myosin‐actin cross‐bridge kinetics via increased estrogen receptor signaling (Kalbe et al., 2007). In vitro, the combination of EE and dienogest (fourth‐generation progestin) can invoke a pro‐myogenic, proliferative response in human myoblasts (Wiik et al., 2005). Both EE and progestins in hormone therapy formulations appear to elicit a separate effect that may be synergistic when combined (Wiik et al., 2005). Progestins may also differ by androgenicity, with first‐ and second‐generations generally being classified as highly androgenic, while third‐ and fourth‐generation progestins are classified as less androgenic (Guo et al., 2010). The androgenicity depends on the parent molecule; for example, 19‐nortestosterone derivatives such as levonorgestrel are derived from testosterone, removing carbon‐19 from and changing the effect of the drug from androgenic to progestogenic, but residual androgenic activity persists, making it an androgenic progestin (Darney, 1995). Drospirenone, however, is structurally derived from spironolactone, but has anti‐androgenic and anti‐mineralocorticoid activity (Sitruk‐Ware, 2004). Androgens play a role in promoting myogenesis, protein synthesis, and muscle repair (Lee et al., 2021; Pieber et al., 2001). Accordingly, the use of specific generations of OCs and their associated progestins may alter the muscle regenerative response in long‐term users.

While training studies may infer a benefit of certain OCs on muscle mass and strength depending on OC type, acute investigations of OC hormones on muscle repair and regeneration are less clear. Wallner and colleagues (Wiik et al., 2005) showed decreased GDF‐8 expression in OC users (EE + dienogest), a molecule that inhibits myogenesis and muscle cell growth (Wiik et al., 2005). Other studies show a greater degree of cell membrane permeability in OC users, suggesting greater release of intramuscular proteins into the circulation and a more robust cytokine response in response to damaging exercise (Ding et al., 2018; Orfanos et al., 2016; Shanmugam et al., 2023). While these studies are informative, very rarely are the specific hormones in OCs reported, and thus, definite conclusions cannot be made about whether OCs are beneficial or detrimental in the post‐exercise muscle response. In particular, the effect of OCs on the myogenic program is important as these drugs may invoke a different systemic milieu that primes the body for contraceptive purposes. Thus, the effects of OC use must be investigated at the cellular level to delineate the hormone‐based differences in muscle damage, repair and regeneration.

In this study, we aimed to investigate the myogenic effects of OC hormones ethinyl estradiol (EE) and progestins medroxyprogesterone acetate (MPA), levonorgestrel (LNG), desogestrel (DSG), and drospirenone (DRP). The primary purpose was to investigate the impact of OC exposure on proliferation, differentiation, migration, and protein abundance in C2C12 mouse myoblasts. We hypothesized that OC formulations would differentially influence each of the above‐mentioned properties based on their distinct progestin profiles. The secondary purpose was to explore the influence of OC hormones on the molecular and cellular responses to damage using electrical pulse stimulation (EPS). Given that androgenic hormones may enhance the myogenic response to damaging exercise, we hypothesized that first‐ and second‐generation OC hormones might elicit a more potent damage and subsequent myogenic response compared to third‐ and fourth‐generation OCs following EPS.

## 2 METHODS

### 2.1 Cell culture

Murine C2C12 skeletal muscle myoblasts from American Type Culture Collection (Cedarlane) were cultured in uncoated 150 mm tissue culture dishes in 5% CO_2_ at 37°C. Cells were grown in phenol red‐free non‐charcoal‐stripped growth media (GM) to eliminate any confounding effect of phenolic compounds (de Leo et al., 2016). The GM contained Dulbecco's modified Eagle's medium (DMEM; Thermo Fisher Scientific, Cat#21063029), with 10% fetal bovine serum (FBS), 1% penicillin/streptomycin (Invitrogen), and 1% sodium pyruvate (Gibco). GM was changed once daily, and cells were passaged at 70% confluency using TrypLE™ Select dissociation reagent (Gibco, Cat#12563029). Differentiation was induced when cells were ~90% confluent with differentiation medium (DM) also composed of phenol red‐free non‐charcoal‐stripped DMEM and supplemented with 2% horse serum (Invitrogen, Cat#16050122) and 1% penicillin/streptomycin. DM was changed every 48 h. All experiments were conducted with cells in passages 6–10. Technical triplicates were averaged to create each N, with a total *N* = 3 per condition and time point.

### 2.2 Dosing protocols

C2C12 myoblasts were seeded onto uncoated 6‐, 24‐ or 96‐well plates (Fisher Scientific, Cat# 087721, 087721B, and VWR, Cat# 82050‐760, respectively) and left to adhere for 24 h prior to treatment. Synthetic hormones ethinyl estradiol (EE; Sigma‐Aldrich, Cat# PHR1480‐200MG), and progestins medroxyprogesterone acetate (MPA; Sigma‐Aldrich, Cat# PHR1589‐500MG), levonorgestrel (LNG; Sigma‐Aldrich, Cat# PHR1850‐500MG), desogestrel (DSG; Sigma‐Aldrich, Cat# PHR1830‐100MG), and drospirenone (DRP; Sigma‐Aldrich, Cat# SML0147‐10MG) were dissolved in nitrogen‐purged dimethyl sulfoxide (DMSO; Thermo Fisher Scientific) at the indicated concentrations. At 60%–70% confluence, cells were treated with the appropriate hormone for baseline concentration assays (EE; 75–150 pg/mL; MPA/LNG/DSG/DRP; 5 ng/mL). Two different EE concentrations were used to replicate the different formulations that exist in high‐ and low‐EE pill formulations. For oral contraceptive generation formulations, higher‐dose EE (150 pg/mL) was combined with either MPA, LNG, DSG, or DRP in the following concentrations: 1st Generation (OC1; EE: 150 pg/mL + MPA: 5 ng/mL), 2nd Generation (OC2; EE: 150 pg/mL + LNG: 5 ng/mL), 3rd Generation (OC3; EE: 150 pg/mL + DSG: 5 ng/mL), and 4th Generation (OC4; EE: 150 pg/mL + DRP: 5 ng/mL). Final concentrations were prepared in fresh GM or DM, and the volume of DMSO did not exceed 0.1% of the total volume of media.

### 2.3 Cell concentration

We evaluated live and total cell counts via trypan blue infiltration into dead cells. Cells were washed twice in 1× phosphate‐buffered saline (PBS) and dissociated using TrypLE™. The cells were then supplemented with an equal volume of GM to neutralize the dissociation media, followed by washes with PBS which were also collected to include dead cells in the count. Once all solution was collected, it was then centrifuged at 200*g* for 5 min. After centrifugation, cell‐free media was aspirated and cells were resuspended in fresh GM, and equal volumes of cell suspension and 0.4% trypan blue (Corning) were mixed together. 10 μL of the final mixture was loaded into each side of a cell counting chamber slide (Invitrogen) and imaged using a Countess automated cell counter (Invitrogen). Live (clear) and dead (blue) cells were counted, and viability was reported as a percentage of live cells relative to the total cell count.

### 2.4 Cell proliferation

We determined cell proliferation using a colorimetric MTT (3‐(4,5‐dimethylthiazol‐2‐yl)‐2,5‐diphenyltetrazolium bromide; Abcam, Cat#AB211091) assay as previously described (Leehy et al., 2017). C2C12 myoblasts were plated in an uncoated 96‐well plate and left to adhere for 24 h. Cells were then treated with the appropriate hormone condition (mentioned above) or DMSO of the same volume for 24 h. After this, media was completely removed and replaced with 20uL of MTT solution (5 mg/mL in 1XPBS; Sigma‐Aldrich) and 180 uL DMEM (final concentration of 0.5 mg/mL MTT solution) per well. Cells were incubated at 37°C and 5% CO_2_ for 3 h. The solution was then carefully removed and replaced with 200 μL of DMSO (used here as MTT/formalin crystal solvent). The plate was then wrapped in foil and placed on a shaker for 15 min, following which the absorbance was measured at 590 nm (Synergy™ Mx, Biotek).

### 2.5 Cell migration

To assess cell migration, we used an in vitro scratch assay. Cells were grown to ~90% confluency in each well of an uncoated 24‐well plate, and a straight line was scratched down the middle. Cells were then washed gently with 1XPBS to remove any dislodged or dead cells in the cleared path. The appropriate conditions in GM were then added to each well, and after imaging, the cells were left to incubate in 37°C and 5% CO_2_ for 8 h then imaged again. An 8‐h window provided a robust dynamic range to detect both accelerations and inhibitions of migration before the scratch wound in the control group began to fully close, which would obscure quantitative differences. Each condition was observed under an inverted microscope, and cell migration was quantified as the percentage of wound‐healing rate (i.e., distance migrated/original wound distance × 100%).

### 2.6 Immunocytochemistry

C2C12 myoblasts were plated in uncoated 24‐well plates and left to adhere for 24 h, after which the appropriate hormone conditions were added (or DMSO as a vehicle control). Cells grew to ~90% confluency and were then washed twice with 1XPBS and switched to DM, which was changed every 48 h. After 6 days of differentiation, myotubes were washed with 1XPBS, then fixed with 4% paraformaldehyde (PFA) for 10 min. Myotubes were washed once more in 1XPBS then permeabilized with 0.1% Triton X‐100 in PBS for 20 min, followed by blocking with 5% goat serum (Sigma‐Aldrich, Cat#G9023‐10ML) in 2% bovine serum albumin (BSA) in PBS for 1 h. Myotubes were incubated in desmin (1:500; ab32362; Abcam) in PBS with 2% BSA overnight at 4°C. The next day, myotubes were washed three times with 1XPBS and incubated with Alexa Fluor® 488 goat anti‐rabbit secondary antibody (Invitrogen, Cat#A11008) at 1:1000 in PBS for 1 h at room temperature. Wells were then washed again with 1XPBS before incubation with 4′,6‐diamidino‐2‐phenylindole (DAPI; Sigma‐ Aldrich, Cat#D9542‐10MG) for 10 min to label nuclei followed by one more wash with 1XPBS. Cover slides were then mounted using a fluorescent mounting medium (DAKO, Cat#S3023). Myotubes were imaged at 20× magnification using an Eclipse Ti2 microscope (Nikon) equipped with DAPI and FITC fluorescence filters. Images were acquired as 16‐bit with a 300 ms exposure time, and were analyzed using NIS Elements (V4.40, Nikon). All quantitative analyses were performed blinded to the treatment condition. Myotube diameter was calculated as the average width of the myotube across 10 equidistant points. Myonuclear index was calculated as the number of nuclei located within myotubes divided by the total number of nuclei in a particular region of interest.

### 2.7 Electrical pulse stimulation

Electrical pulse stimulation (EPS) was used to elicit contractile stress to myotubes after 6 days of differentiation, adapted from a protocol by Orfanos et al. (2016). Each condition was grown in 6‐well plates and using the C‐Pace EM (IonOptix) myotubes were stimulated at 23 V with a 15 Hz pulse for 5 s and 5 Hz pulse for 5 s, separated by a 5 s break. After 3 h of EPS, electrodes were removed, and myotubes were either collected immediately (3hS) or after 12 h (3 + 12). Control groups underwent the same intervention without stimulation (i.e. collected at baseline or collected after 3 h incubation, NS).

### 2.8 Protein extraction and immunoblotting

Myotubes were lysed with a combination of ice‐cold Radioimmunoprecipitation assay buffer (RIPA; Sigma‐Aldrich, Cat#R0278) and Halt protease inhibitor and phosphatase inhibitor (Thermo Fisher Scientific, Cat#78446). Samples were scraped and incubated on ice for 30 min, then spun at 11,000 *g* for 10 min at 4°C to pellet debris. Supernatants were collected and stored at −80°C. Protein concentrations were measured using a bicinchoninic acid (BCA) protein assay (Thermo Fisher Scientific, Cat#23225). Samples were denatured in sodium dodecyl sulfate (SDS) sample buffer (60 mmol/L Tris pH 6.8, 25% glycerol, 2% SDS, 10% 2‐mercaptoethanol, and 0.1% Bromophenol Blue) for 5 min at 95°C. 10 ug of each sample was loaded into individual lanes of a 4%–15% Criterion TGX precast protein gel (Bio‐Rad, Hercules, CA, USA). Electrophoresis was performed at 200 V for 45 min at room temperature. Proteins were then transferred onto a nitrocellulose membrane (Bio‐Rad, Cat#1620112) by turbo transfer. Membranes were stained with Ponceau S solution (Sigma‐Aldrich) for confirmation of equal protein loading, then blocked with 5% bovine serum albumin (BioShop Canada, Cat#ALB007.500) for 90 min at room temperature. After blocking, membranes were incubated overnight 4°C in the respective primary antibody at 1:1000 for Calpain 3 (sc‐365,277); HSP70 (sc‐24); MyoD (sc‐377,460); ERα (sc‐787) (Kalbe et al., 2007; Wiik et al., 2005); and ERβ (sc‐53,494) (Guo et al., 2010; Lee et al., 2021); 1:500 for PRα/β (sc‐810) (Pieber et al., 2001; Shanmugam et al., 2023); and 1:100 for Myogenin (sc‐12,732); purchased from Santa Cruz Biotechnology, Santa Cruz, CA. Following three Tris‐buffered saline and Tween‐20 (TBST) washes, membranes were incubated in the appropriate secondary antibody (Cell Signaling Technologies, Cat#7074, #7076) at 1:3000 in 5% BSA in TBST for 90 min at room temperature. Proteins of interest were detected by chemiluminescence solution (Clarity Western ECL substrate, Bio‐Rad, Cat#1705062) using the ChemiDoc MP Imaging System and analyzed using the Image Lab Software 6.0.1. Immunoblot images are available in Data S1 (https://doi.org/10.6084/m9.figshare.30826949).

### 2.9 Creatine kinase assays

Creatine kinase (CK) was analyzed via CK‐NAC colorimetric assay (MTI Diagnostics, Cat#552‐530) per manufacturer instructions. Media from each condition within each intervention was collected, centrifuged at 200 g for 5 min to pellet cellular debris, and the supernatant was collected and stored at −80°C until tested.

### 2.10 Statistical analyses

All statistical analyses were performed using Prism (V9.0.1, GraphPad Software LLC). Sample sizes were based on previously established hormone exposure and EPS models (e.g. Orfanos et al. (2016); Ding et al. (2018)). For statistical comparison of multiple groups, we utilized a one‐way or two‐way analysis of variance (ANOVA), with hormone and time (0 h, 3 h post‐EPS, and 12 h post‐EPS) as the two‐way factors. Where a significant interaction was detected, a Bonferroni post hoc test was performed. Statistical significance was set to *p* < 0.05. Data are presented as means ± SD.

## 3 RESULTS

Tables 1a and 1b provide a comprehensive summary of all measured outcomes.

**TABLE 1a:** Summary of findings for proliferation, migration, and differentiation (myotube diameter, myonuclear index).

Hormone/outcome		Proliferation	Migration	Differentiation parameters	
				Myotube diameter	Myonuclear index
EE	75	↔	↔	↓	↑
	150	↔	↔	↔	↔
PROG	MPA	↑	↑	↔	↔
	LNG	↑	↔	↔	↔
	DSG	↔	↔	↔	↑
	DRP	↔	↔	↔	↑
OC	1	↓	↓	↔	↔
	2	↓	↓	↔	↔
	3	↔	↓	↔	↔
	4	↔	↓	↔	↔

**TABLE 1b:** Summary of findings for creatine kinase concentration (U/L) and protein content (Calpain 3, HSP70, MyoD, Myogenin, ERα, ERβ, PRα, PRβ).

Hormone/outcome		CK (U/L)			Protein content																							
					Cal3			HSP70			MyoD			MyoG			ERα			ERβ			PRα			PRβ		
		NS	S	S + 12	NS	S	S + 12	NS	S	S + 12	NS	S	S + 12	NS	S	S + 12	NS	S	S + 12	NS	S	S + 12	NS	S	S + 12	NS	S	S + 12
VEH		\	↑	↓	\	↑	↔	\	↑	↑↑	\	↔	↔	\	↓	↔	\	↔	↓	\	↔	↑	\	↑	↑	\	↓	↓
OC	1	↓	↓↑	↑ ↑	↑	↔↔	↑ ↔	↔	↔↑	↑ ↑↑	↑	↔↓	↔↓	↔	↓↓	↓↔	↓	↓↓	↑ ↑	↑	↑ ↓	↔↓	↑	↑ ↔	↔↔	↑	↑ ↔	↑ ↑
	2	↓	↓↑	↑ ↑	↑	↔↔	↑ ↔	↑	↑ ↑	↑ ↑↑	↑	↑ ↓	↓↓↓	↔	↑ ↔	↓↓	↑	↓↓	↑ ↔	↑	↑ ↑	↔↔	↑	↑ ↑	↑ ↔	↑	↑ ↓↓	↑ ↓
	3	↓	↓↔	↓↔	↓	↔↑	↔↑	↑	↔↑	↑ ↑↑	↑	↑ ↓	↔↓↓	↑	↑ ↔	↑ ↔	↑	↔↔	↑ ↓	↔	↔↔	↔↑	↑	↔↔	↑ ↑	↑	↑ ↓	↑ ↓↓
	4	↓	↓↔	↓↓	↓	↓↔	↔↔	↑	↑ ↑	↑ ↑↑	↑	↔↓	↑ ↔	↑	↑ ↓	↑ ↔	↑	↑ ↓	↑ ↔	↑	↑ ↔	↔↔	↑	↓↔	↔↑	↑	↑ ↔	↑ ↔

*Note*: Conditions: NS = No EPS, S = EPS (3 h), S + 12 = EPS (3 h) + 12 h incubation. Black arrows = relative to NS condition (within a hormone condition). Colored arrows = relative to vehicle condition (within a time point).

### 3.1 Synthetic progestins differentially affect myoblast proliferation and migration

**FIGURE 1:**
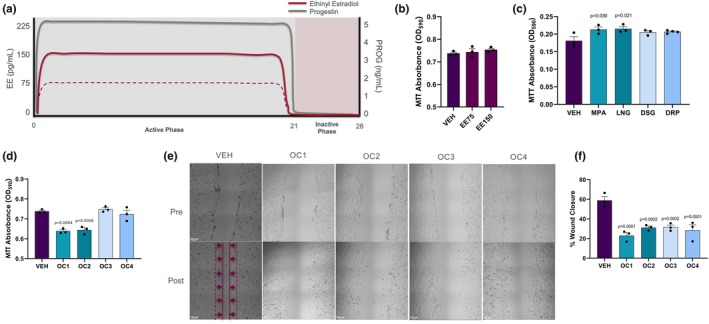
(a) Schematic of the oral contraceptive cycle (OC), with ethinyl estradiol (EE) depicted by a red line (solid = 150 pg/mL, dashed = 75 pg/mL) and progestins (PROG) depicted by a gray line. (b) MTT absorbance (indicative of proliferation) after EE dosing. (c) MTT absorbance (indicative of proliferation) after progestin dosing (MPA, medroxyprogesterone acetate; LNG, levonorgestrel; DSG, desogestrel; DRP, drospirenone). (d) MTT absorbance (indicative of proliferation) with OC formulation dosing. OC1 = First generation (EE + MPA), OC2 = Second generation (EE + LNG), OC3 = Third generation (EE + DSG), OC4 = Fourth generation (EE + DRP). (e) Visual representations of scratch assay for VEH, OC1, OC2, OC3 and OC4 conditions quantified in (f). Post represents 8 h after scratch. Red dotted lines indicate wound borders at time 0, solid lines indicate wound borders 8 h post‐scratch. (f) Percent wound closure after scratch assay with OC phase dosing. Statistical comparisons were performed using a one‐way ANOVA. *p*‐value indicates a significant difference from vehicle condition (no hormone; VEH).

During a typical cycle of OC use, the concentration of ethinyl estradiol (EE) and progestin will go through high‐ and low‐hormone phases, ranging from ~0 to 150 pg/mL of EE and ~0–5 ng/mL of progestins (Figure 1a) (Allen, 2022). To examine if these drugs can affect myoblast dynamics, C2C12 cells were treated for 24 h with each synthetic OC hormone at average serum concentrations observed in OC users. EE at either 75 or 150 pg/mL had no impact (Figure 1b), but progestins MPA and LNG were capable of independently stimulating proliferation (*p* < 0.05, Figure 1c). OC formulations of first‐ and second‐generation contraceptives (OC1 [EE + MPA] and OC2 [EE + LNG]) significantly decreased proliferation, while third‐ and fourth‐generations [OC3 (EE + DSG) and OC4 (EE + DRP)] did not differ from the vehicle control (VEH; Figure 1d).

Next, we assessed cellular migration as an indicator of the mobility of myoblasts in response to injury (Chazaud, 2020; Tidball, 2005). A scratch wound was applied to C2C12 myoblasts followed by an 8 h treatment with EE, progestin (MPA, LNG, DSG, DRP) or OC formulations (OC1, OC2, OC3, OC4) (Figure 1e). Migration was evaluated based on the percentage of wound closure after 8 h of treatment (Post) relative to the initial scratch (Pre). The independent dosing of EE or progestins had no effect on cell migration, apart from MPA, which significantly increased the rate of wound closure (*p* < 0.05; Figure S1A,B). All OC generations significantly impaired myoblast migration [OC1 (61%; *p* < 0.001), OC2 (47%; *p* < 0.001), OC3 (46%; *p* < 0.001), and OC4 (52%; *p* < 0.001)] compared to VEH treatment (*p* < 0.05; Figure 1f).

### 3.2 EE and progestins, but not OC formulations, alter myotube growth

**FIGURE 2:**
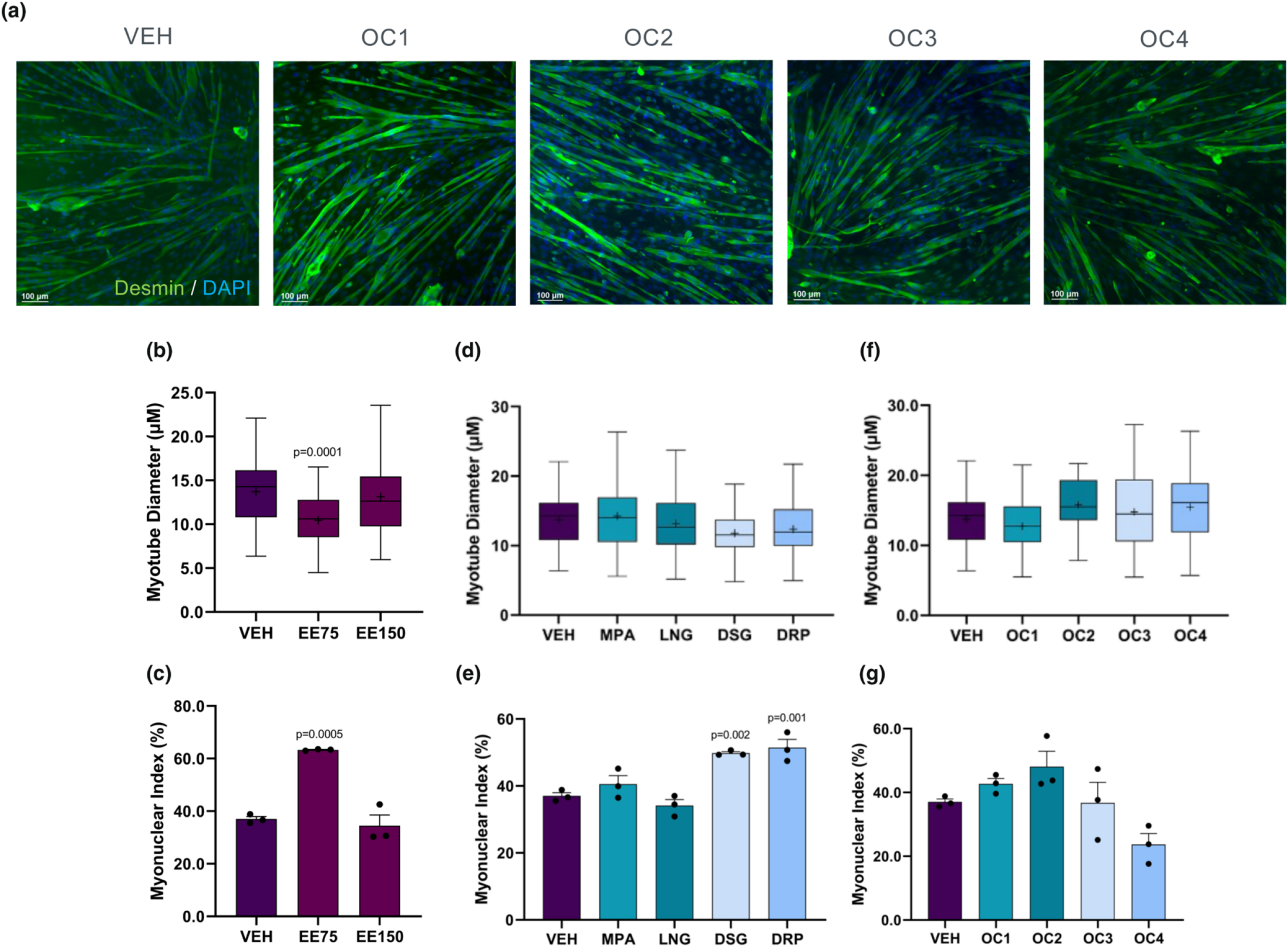
(a) Representative images of C2C12 myotubes 6 days after differentiation in vehicle (VEH), OC1, OC2, OC3, and OC4 formulations. Myotubes were stained with desmin (green) and DAPI (blue). (b, d, f) Myotube diameter (um) of vehicle, EE, progestin, and OC formulations after 6 days of differentiation. (c, e, g) Myonuclear index of vehicle, EE, progestin and OC formulations after 6 days of differentiation. Values are presented as median lines and interquartile range (boxes) ± maximum and minimum values (whiskers), with + representing the mean. Statistical comparisons were performed using a one‐way ANOVA. *p*‐value indicates a significant difference from vehicle (VEH).

We next evaluated the effect of each hormone on C2C12 myotube diameter and myonuclear index as indicators of myotube differentiation (Figure 2a) (Bajaj et al., 2011). Myoblasts were treated with either EE or progestins for 6 days in low serum conditions to induce differentiation. After 6 days of differentiation, myotubes were stained for desmin and DAPI, and myotube diameter and myonuclear index were evaluated. EE at a low dose (i.e., EE75) showed significantly lower myotube diameter but higher myonuclear index (*p* < 0.05), while EE150 did not influence either myotube diameter or myonuclear index (Figure 2b,c). The independent treatment of differentiating myoblasts with progestins had no impact on myotube diameter, but DSG and DRP were able to significantly elevate myonuclear index (*p* < 0.05; Figure 2d,e). When combined with EE to simulate OC conditions, no progestins were capable of altering myotube differentiation (Figure 2f,g).

### 3.3 OC generation differentially impacts hormone signaling, myogenesis, and the stress response to EPS

**FIGURE 3:**
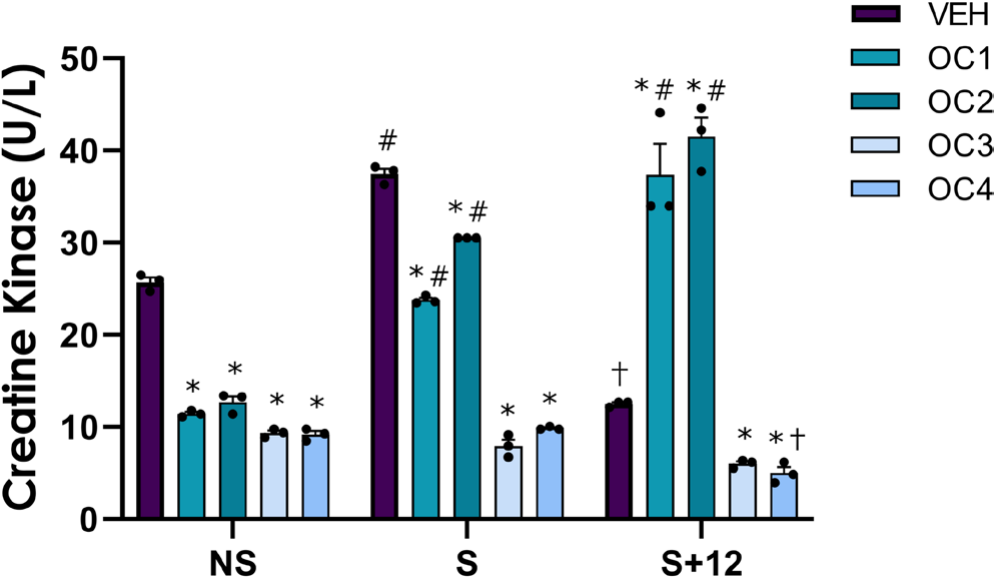
Creatine kinase response to vehicle (VEH), OC1, OC2, OC3, and OC4 treatment with no electrical pulse stimulation (EPS) as no stim (NS); 3 h of EPS (S), and 3 h of EPS with 12 h of further incubation (S + 12). Statistical comparisons were performed using a two‐way ANOVA (Factors: Time and hormone condition). *Indicates a significant difference (*p* < 0.05) from vehicle (VEH). #Significantly higher (*p* < 0.05) than NS within hormone condition. †Significantly lower (*p* < 0.05) than NS within hormone condition.

Given the observed change in CK levels with OC treatment (Figure 3 – NS conditions), we then aimed to investigate the molecular response following electrical pulse stimulation (EPS) to determine whether OCs can influence the stress and repair mechanisms of myotubes. All conditions were set to the same EPS conditions (23 V, 15 Hz pulse for 5 s and 5 Hz pulse for 5 s, 5 s delay), with no observed differences in the intensity or synchronicity of contractions between the different hormone conditions. We first measured creatine kinase (CK) concentration in the conditioned media of hormone‐treated myotubes as an indicator of myotube membrane damage (Collins et al., 2019; Kendall & Eston, 2002; Persky et al., 2000). In response to EPS (timepoint “S”), first‐ and second‐generation OC formulations exhibited significant increases in CK concentrations, which increased further after 12 h (timepoint “S + 12”; *p* < 0.05; Figure 3). Third‐ and fourth‐generation OC formulations severely blunted this increase in CK, particularly reducing CK concentrations in fourth‐generation OC formulations 12 h post‐EPS (*p* < 0.05; Figure 3).

**FIGURE 4:**
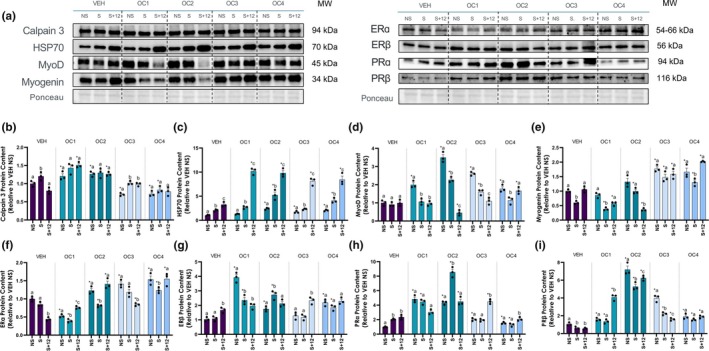
Protein content in response to OC dosing; OC1 = First generation (EE + MPA), OC2 = Second generation (EE + LNG), OC3 = Third generation (EE + DSG), OC4 = Fourth generation (EE + DRP). (a) Representative western blots of Calpain 3, HSP70, MyoD, Myogenin, ERα, ERβ, PRα, and PRβ. NS = No EPS, S = EPS (3 h), S + 12 = EPS (3 h) + 12 h incubation. Ponceau S staining is shown below to confirm equal loading across all samples. Graphical quantification of (b) Calpain 3, (c) HSP70, (d) MyoD, (e) Myogenin, (f) ERα, (g) ERβ, (h) PRα, and (i) PRβ protein content. Data are means ± SD. Statistical comparisons were performed using a two‐way ANOVA (Factors: Time and hormone condition). Where an interaction was detected, letters were used to denote differences between time points within a hormone condition. Time points that do not share a letter are significantly different (*p* < 0.05). *denotes a significant difference (*p* < 0.05) from VEH within a time point.

Next, we measured Calpain 3, a protein involved in proteolysis and structural remodeling (Beckmann & Spencer, 2008), using western blotting (Figure 4a). Immediately following EPS, Calpain 3 protein content significantly increased but returned to baseline by 12 h post‐stim (*p* < 0.05; Figure 4b). OC1 and OC2 dosing significantly increased Calpain 3 content with no EPS (timepoint “NS”), which remained elevated following EPS. OC3 and OC4, however, exhibited significantly reduced Calpain 3 content at NS, with transient increases in OC3 but no change in OC4. HSP70 had a stepwise increase in protein content from NS to S + 12 under all hormone conditions (*p* < 0.05; Figure 4c). OC2 and OC4 had a larger increase in HSP70 content at S, but all OC generations experienced an augmented rise by S + 12. EPS did not affect MyoD protein levels under VEH conditions, but it was significantly altered by OC treatment (*p* < 0.05; Figure 4d). OC use significantly increased MyoD protein content at baseline, which decreased at the S timepoint; OC2 and OC3 further decreased MyoD at S + 12. Meanwhile, VEH‐treated myotubes experienced a transient decrease in myogenin protein content at S that returned to baseline levels 12 h later (*p* < 0.05; Figure 4e). This pattern was consistent in the OC1 and OC4 conditions, but OC2 reduced myogenin content at S and even more at S + 12. OC3‐ and OC4‐treated myotubes had significantly elevated myogenin protein relative to VEH at all time points.

To elucidate potential pathways by which EE and progestins may exert their effect, we looked at sex hormone receptor content in response to hormone dosing and EPS. Interestingly, hormone receptor isoforms (ERα/β, PRα/β) exhibited alternative responses to EPS: At S + 12, ERα was significantly lower while ERβ had increased; at S and S + 12, PRα was elevated while PRβ was inhibited (*p* < 0.05; Figure 4f–i). However, when myotubes were grown with OC treatment, this pattern changed. With OC1, OC2, and OC4 treatment, ERα protein content decreased from NS to S before rising again at S + 12. OC3, on the other hand, had a progressive decline in ERα protein at S and S + 12. Curiously, the second‐ to fourth‐ generation OCs seemed to elevate ERα levels at NS and S + 12, while OC1 appeared to decrease it at almost every timepoint. ERβ was significantly elevated at baseline in OC1, OC2 and OC4 relative to VEH‐treated C2C12 myoblasts. At S, ERβ protein content decreased with OC1 but increased with OC2, with no change with OC3/4 treatment. 12 h post‐EPS, ERβ had returned to NS levels with OC2/4, while OC1 remained unchanged and OC3 increased. PRα protein content was significantly upregulated in all OC conditions at NS. At S, only treatment with OC2 elevated PRα levels, which decreased back to baseline at S + 12. Finally, OC3/4 conditions had increased PRα content at S + 12. OC use significantly increased PRβ protein content across most timepoints, regardless of generation. With EPS, OC1 had a delayed rise in PRβ that only occurred at S + 12. OC2 and OC3 exhibited a decrease in PRβ content at S relative to NS, which was restored only in the OC2 condition. Lastly, OC4 treatment blunted any effect of EPS on PRβ protein levels.

## 4 DISCUSSION

The purpose of this study was to investigate the impact of synthetic female sex hormone exposure on murine C2C12 myoblast function and on the response to EPS. While earlier studies have tested synthetic forms of estrogen (namely, 17α‐ethinyl estradiol), hormones belonging to OC formulations in isolation and in combination have not been assessed for their response to contractile stress. We found that first‐ and second‐generation OC formulations decreased proliferation while all formulations decreased migration; however, isolated hormones (i.e., EE alone or progestins alone) exerted a different effect. Despite lower baseline CK compared to VEH, OC1 and OC2 formulations exhibited a profound CK response to EPS compared to OC3 and OC4. Earlier generation OC treatment upregulated PR content, which may be linked to the observed changes in myogenic regulatory factors.

Our findings indicate a complex interplay between EE and various progestins in modulating cell proliferation, differentiation, and migration, expanding upon existing literature that highlights the distinct effects of hormone interactions. Specifically, we observed an increase in proliferation in first‐ and second‐generation progestins alone, but a decrease in proliferation when these progestins were combined with EE (Figure 1c,d). This result suggests that the combination of EE with these specific progestins may induce a regulatory mechanism that counteracts the proliferative effects seen with the progestins alone, also known as hormonal antagonism or negative synergism. Alternatively, we observed synergistic effects of EE + third‐generation progestins, whereby the combination of the two reduced migration significantly (Figure 1f). Both cases may be depictions of hormone permissiveness, whereby a hormone cannot exert its full effects without the presence of another hormone (Malbon et al., 1988). Impaired migration may hinder the ability of muscle precursor cells to reach sites of injury, potentially delaying muscle regeneration. Fourth‐generation progestins such as dienogest have been shown to elicit pro‐myogenic and proliferative effects in human skeletal muscle cells due to their relatively low binding affinity to the progesterone receptor and low androgenicity (Regidor & Schindler, 2017; Wiik et al., 2005). However, our experiments using drospirenone, another fourth‐generation progestin, did not demonstrate functional benefits in myoblasts, such as increased proliferation, differentiation, or migration. Drospirenone differs from dienogest in its anti‐mineralocorticoid activity, partially counteracting estrogen‐induced changes in protein abundance where dienogest does not (Kuhl, 1996). This distinction highlights the importance of the specific progestin used despite belonging to the same generation. The varying degrees of androgenicity ultimately depend on the precursor of the progestin and the nature of its structural changes (Darney, 1995; de Leo et al., 2016; Sitruk‐Ware, 2004).

The current investigation unveiled a differential response among OC formulations, particularly between OC1/OC2 and OC3/OC4 formulations. This observation was seen most notably in the significantly upregulated CK response to EPS in OC1 and OC2 formulations and concomitant blunting of the CK response to EPS in OC3 and OC4 formulations. CK is a known marker of cell membrane stress and muscle cell differentiation in vitro (Ito et al., 2001), therefore this result suggests that these formulations may heighten membrane susceptibility to mechanical stress, potentially influencing muscle repair processes. A key difference between OC1/OC2 and OC3/OC4 formulations is the androgenicity of the progestins, potentially driving the enhanced CK responses to EPS observed in the present study. Previous literature investigating OC use in humans has shown greater increases in serum CK in OC users (Orfanos et al., 2016; Roth et al., 2001; Shanmugam et al., 2023), while others have not (Ding et al., 2018; Savage & Clarkson, 2002). This discrepancy may be a direct result of testing individuals using OCs without controlling for OC type, as well as a lack of reporting of the generations and specific progestins consumed by participants. Androgenicity alone, however, may not fully account for the divergent effects of synthetic progestins on contraction‐related muscle stress. While the ability of progestins to differentially impact membrane integrity is yet to be elucidated, the finding of greater suppression of CK release with OC3 and OC4 formulations may reflect a combination of factors: progestin‐specific PR isoform signaling, differences in off‐target receptor binding, progestin‐specific ER modulation, and/or non‐genomic membrane interactions and metabolite profiles (Leehy et al., 2017; Singhal et al., 2016; Sitruk‐Ware, 2004). Thus, the net effect of a given progestin on membrane integrity may arise from the integrated contribution of these properties rather than androgenicity alone. Nevertheless, increased CK expression has been shown to increase insulin‐like growth factor 1 (IGF‐1) expression and facilitate the monocyte‐to‐macrophage transition (Loike et al., 1984) , which points to its utility in muscle repair/regeneration and aligns well with our findings that an increase in CK expression is related to early myogenic regulatory factor protein content (Zanou & Gailly, 2013).

MyoD plays a pivotal role in the earlier stages of myogenesis, crucial to myogenic determination in directing myoblast fusion into multinucleated primary myofibers (Zammit, 2017). In the current investigation, we observed considerably greater MyoD protein content with OC2 treatment at NS and S, suggesting that OC2 treatment may influence early‐stage myogenesis. OC2 formulations may prime muscle cells for differentiation, potentially affecting muscle regeneration efficiency. In contrast, OC3 and OC4 treatment may primarily affect late‐stage myogenesis, as evidenced by greater myogenin protein content at all time points. Furthermore, androgen receptors are more highly expressed during C2C12 differentiation, providing an environment in which androgenic hormones may thrive and exert their effects more robustly (Wannenes et al., 2008). This may explain our observation of greater MyoD protein content in OC2, as well as the lack of proliferation and migration in OC1 and OC2 formulations, as these cells are undifferentiated and thus express fewer androgen receptors by which OC1 and OC2 hormones may exert their effects. These results suggest that the androgenic properties of certain progestins in combination with EE may robustly impact cellular and molecular responses, highlighting the importance of considering hormone formulations and generations of OCs in the context of contractile stress, repair and regeneration.

Alongside higher CK in OC1 and OC2 formulations, we observed a generation‐specific Calpain 3 response to hormone dosing and EPS. Calpain 3 is responsible for the structural organization, maintenance, and turnover of sarcomeres, essential for the early adaptive response to damaging exercise (Beckmann & Spencer, 2008). Calpain 3 was upregulated with OC1 and OC2 treatment yet unchanged with EPS. This outcome suggests a potential phenotypic change in baseline structural turnover that negates the need for further upregulation with EPS. OC3 and OC4 downregulated Calpain 3 at baseline, only increasing with OC3 treatment after EPS to levels similar to VEH. Paired with the absence of a CK response as an indicator of myotube lesion formation, this may suggest a reduced requirement for structural remodeling in the OC3 and OC4 conditions. Calpain 3 not only assists in removing damaged proteins and cellular debris, aiding in the transition from the damage phase to the repair phase but is also closely related to SC self‐renewal, which occurs during muscle regeneration (Stuelsatz et al., 2010). Calpain 3 also downregulates MyoD expression in order to aid in promoting the establishment of a pool of reserve cells necessary for self‐renewal (Stuelsatz et al., 2010), which may explain the decrease in MyoD with EPS in OC1, OC2 and OC3 conditions and 12 h after all treatments. This suggests that earlier generation OCs may promote more active muscle remodeling, while later generation OCs may exert a stabilizing effect. Altogether, this highlights the potential role of specific OC formulations in mitigating contractile stress, regulating myogenesis and aiding in the re‐establishment of reserve cell populations in C2C12 culture.

HSP70 is also well‐documented for its protective effects against muscle damage, its role in facilitating repair and recovery, and its contribution to the maintenance of skeletal muscle mass and integrity (Senf 2013). In the current investigation we observed an increase in baseline HSP70 protein abundance with OC2, OC3 and OC4 formulations, suggesting a heightened baseline protective mechanism at play within C2C12 myotubes. In all conditions, however, HSP70 protein content increased with EPS and further increased 12 h after, with no difference between OC conditions at 12 h. This enhanced abundance may point to greater muscle repair and maintenance with OC treatment, aligning with our observation of upregulated myogenic protein content compared to VEH with all OC generations for MyoD and with OC3 and OC4 for Myogenin. Importantly, HSP70 experienced greater upregulation immediately after EPS with OC2 treatment, which pairs with higher MyoD protein abundance at the same time point compared to all other conditions. Since HSP70 is critical for cellular stress protection, this suggests that OC2 may enhance the muscle's ability to cope with mechanical damage, potentially offering protective benefits after high‐intensity efforts. OC3 and OC4, on the other hand, exhibited greater upregulation of myogenin protein content at all time points, which points to potential effects on late‐stage myogenesis in these formulations after damaging stimuli. Altogether, the interplay of HSP70 and myogenic regulatory proteins suggests that the specific generation of OC may target distinct phases of the myogenic program, influencing specific aspects of adaptation and repair.

We observed significant changes in estrogen and progesterone receptor isoforms (α and β) with OC dosing and EPS, suggesting that the regenerative response in OC1 and OC2 may be linked to ER‐ and PR‐mediated mechanisms. In the current investigation, OC1 and OC2 exhibited substantial increases in PRα and PRβ content with hormone dosing and EPS compared to OC3 and OC4. Progesterone receptors α and β may regulate particular genes or proteins synergistically, antagonistically, or with one isoform more strongly regulating an outcome or function than its counterpart (Jacobsen & Horwitz, 2012). As it pertains to myogenic proteins, there is no current literature that pinpoints whether one isoform predominates; thus, this is the first report of progesterone receptor changes in response to four generations of synthetic OC hormone dosing and EPS. It may be theorized, then, that the regulation of proteolytic and myogenic protein content by OC1 and OC2 formulations observed in these experiments may be linked to changes in PR content. Similarly, ethinyl estradiol, the synthetic compound of estrogen in all four generations of OCs, has a greater relative binding affinity for ER, approximately 190% of estradiol's affinity (Blair, 2000). This finding suggests that if functionality is highly mediated by the presence and binding of the hormone to its receptor, more pronounced effects may be seen with synthetic hormone dosing. EE binds more strongly to ERα, which is necessary for SC pool self‐renewal (Collins et al., 2019), but recent reports indicate a predominance of ERβ protein abundance in skeletal muscle (Seko et al., 2020). In the current investigation, we observed higher responsivity of ERβ compared to ERα with hormone exposure in OC1, OC2 and OC4 conditions but greater upregulation of ERα protein content 12 h post‐EPS in OC2 and OC4 with no change or a decrease in ERβ across all OC generations but OC3. Given that OC generations vary in the type of progestin they contain and that the regulation of ER and PR is dependent on both EE and the specific progestin included, this may point to combined hormone effects on myogenic, calpain and sex hormone receptor protein abundance. While EE binds similarly to ERs regardless of generation, each progestin used in these experiments (MPA, LNG, DSG, DRP) has a specific binding affinity to PR (Collins, 1994, Krattenmacher, 2000), which may in part be dictating these responses. Ultimately, however, we are uncertain of the nature of the interactions between EE and each progestin, which may regulate the functions observed in the current investigation. Overall, different OC generations may uniquely influence muscle function through hormone‐specific pathways, which could have implications for long‐term muscle health, training adaptations, and recovery rates in OC users.

This study is not without its limitations. The utilization of the murine C2C12 cell line bears strengths in its ability to study myoblast differentiation and repair, but future studies should consider primary murine or human skeletal muscle cells as well as in vivo experiments. Further experimentation using direct cell counting methods would be an ideal complement to our MTT data to further strengthen our proliferation data. Secondly, our use of non‐charcoal‐stripped media allowed for residual E2 to be present in media; however, this concentration is unlikely to have significantly altered the dose–response relationships reported in this investigation. Another limitation is that the use of EPS may not provide an accurate representation of damaging exercise in humans, as the stimulus and cellular milieu are limited as compared to the relatively complete damage response in humans. More work is needed to provide mechanistic insight into the basic functional changes seen with hormone dosing to help identify key molecules that may encourage or inhibit myogenic regulation prior to and after models of exercise.

## 5 CONCLUSION

Ours is the first study to observe the in vitro effects of differing generations of synthetic hormones on the C2C12 response to contractile stress. Our findings suggest that the progestin type in contraceptive formulations is of importance in muscle cell function, which may have implications on the muscle physiological response and health outcomes in OC users. Future studies should investigate these hormones using human models to further understand the underlying mechanisms at play in response to contractile or mechanical stress. The implications of this will allow for greater consideration of OC formulations in clinical prescriptions and further optimization of hormone therapies to achieve optimal clinical effects of hormonal contraception.

## AUTHOR CONTRIBUTIONS

**Mai Wageh:** Conceptualization; data curation; formal analysis; investigation; methodology; project administration; resources; software; supervision; validation; visualization. **Michael Kamal:** Validation; visualization. **Gianni Parise:** Conceptualization; data curation; formal analysis; funding acquisition; investigation; methodology; project administration; resources; software; supervision; validation; visualization.

## FUNDING INFORMATION

No funding was required to directly support this article. However, much of the work cited from our group was supported by grants from the Canadian Natural Sciences and Engineering Research Council (NSERC).

## CONFLICT OF INTEREST STATEMENT

None of the authors have any relevant conflicts of interest or financial ties to disclose.

## ETHICS STATEMENT

All experiments described in this manuscript were conducted using the C2C12 cell line and therefore ethical approval was not required.

## Supporting information


**Figure S1:** Percent wound closure after scratch assay with (A) EE and (B) progestin dosing. *Indicates a significant difference (*p* < 0.05) from vehicle condition (no hormone; VEH).

**Data S1:** Western Blots.

## Data Availability

The data that support the findings of this study are available from the main author upon reasonable request.
