# Supplementary material for: Synthetic estrogen and progestin effects on the myogenic program following damage in C2C12 murine myoblasts
Source: Physiol Rep. 2026 Apr 26;14(8):e70886. doi: 10.14814/phy2.70886 (PMC13111148; doi:10.14814/phy2.70886)
Supplement: Supplementary file 1 — Figure S1: Percent wound closure after scratch assay with (A) EE and (B) progestin dosing. *Indicates a significant difference (p < 0.05) from vehicle condition (no hormone; VEH). [file PHY2-14-e70886-s001.pdf]

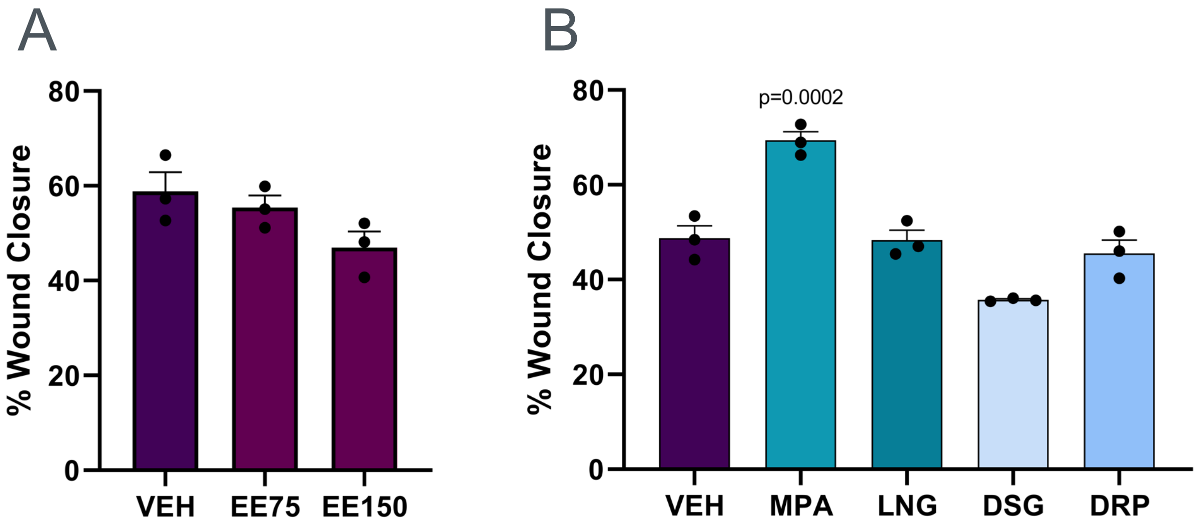

Supplementary Figure S1. Percent wound closure after scratch assay with A) EE and B) progestin dosing. *indicates a significant difference ($p<0.05$) from vehicle condition (no hormone; VEH).
