# Supplementary material for: Synthetic estrogen and progestin effects on the myogenic program following damage in C2C12 murine myoblasts
Source: Physiol Rep. 2026 Apr 26;14(8):e70886. doi: 10.14814/phy2.70886 (PMC13111148; doi:10.14814/phy2.70886)

**Western Blots:**

Below are complete blots for the following proteins: Calpain 3, HSP70, MyoD, Myogenin, ERα, ERβ, PRα, and PRβ. Molecular weights in red indicate migration positions of marker ladder proteins. Molecular weights in blue indicate migration positions of proteins of interest.

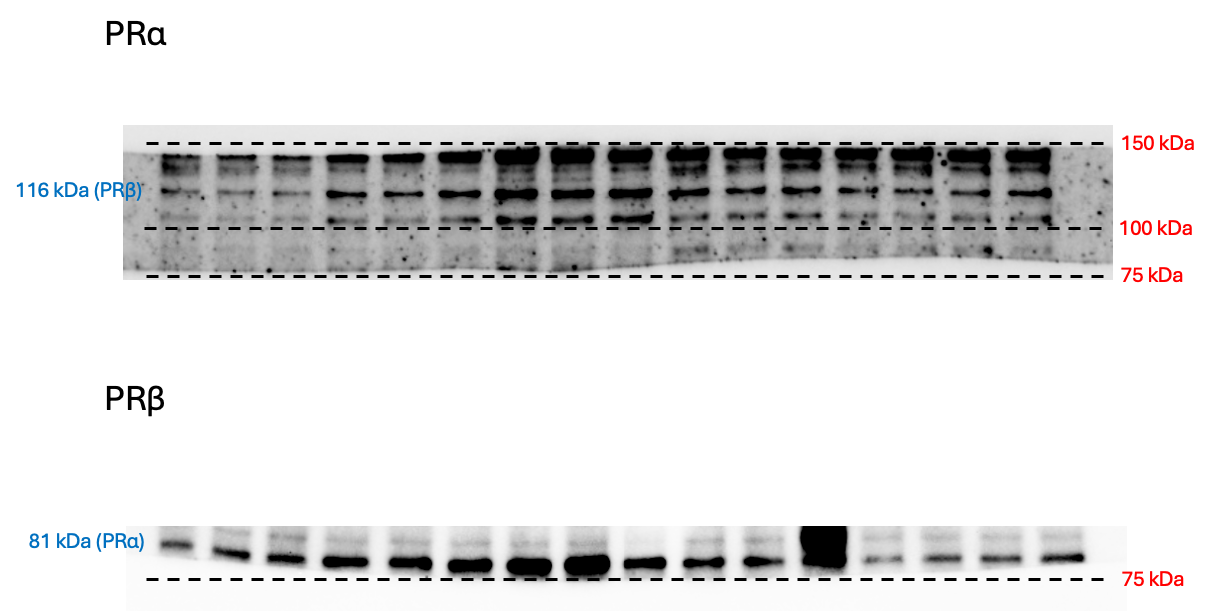

Supplement: Supplementary file 2 — Data S1: Western Blots. [file PHY2-14-e70886-s002.docx]
